# Development of a trispecific fusion protein based on angiotensin-converting enzyme 2, glycoprotein 130, and tumor necrosis factor receptor 2 as a promising therapeutic for COVID-19

**DOI:** 10.1186/s43556-025-00320-4

**Published:** 2025-10-15

**Authors:** Yongfeng Qiao, Yanjun Han, Lu Zhao, Wenjing Gao, Hong Hu, Chao Su, Anqi Zheng, Junqing Sun, Mingxiong Tian, Yarong Wu, Lianmei Bai, Yuping Lei, Jiahao Wu, Weibing Zhang, Pu Han, Xiaoyu Li, Chunbo Dong, Haidong Wang, Zhida Liu, Pengcheng Han

**Affiliations:** 1 College of Veterinary Medicine, Shanxi Agricultural University, Jinzhong, China; 2 Shanxi Academy of Advanced Research and Innovation, Taiyuan, China; 3 Jiangsu Provincial Key Laboratory of Critical Care Medicine, School of Medicine, Zhongda Hospital, Advanced Institute for Life and Health, Southeast University, Nanjing, 210009 China; 4 CAS Key Laboratory of Pathogen Microbiology and Immunology, Institute of Microbiology, Chinese Academy of Sciences, Beijing, China; 5 Ankerui (Shanxi) Biological Cell Co., Ltd., Taiyuan, China; 6 Shanxi Animal Disease Prevention and Control Center, Taiyuan, China; 7 MOE Key Laboratory of Coal Environmental Pathogenicity and Prevention, Shanxi Medical University, Taiyuan, China

**Keywords:** Severe acute respiratory syndrome coronavirus 2, Cytokine storms, Angiotensin-converting enzyme 2, Tumor necrosis factor receptor 2, Glycoprotein 130

## Abstract

Despite a substantial reduction in the incidence of coronavirus disease 2019 (COVID-19) infections, severe cases continue to pose a significant clinical burden, particularly among elderly individuals and patients with underlying medical conditions, due to high viral loads and cytokine storm syndrome. Elevated levels of interleukin-6 (IL-6) and tumor necrosis factor (TNF), signaling through their respective receptors, glycoprotein 130 (GP130)/interleukin-6 receptor (IL-6R) and tumor necrosis factor receptor 2 (TNFR2), are independent predictors of disease severity and mortality. To address this challenge, a series of bifunctional and trifunctional decoy receptor fusion proteins were developed by fusing the extracellular domains of TNFR2 and/or GP130 to an engineered angiotensin-converting enzyme 2 (ACE2) protein, the entry receptor for severe acute respiratory syndrome coronavirus 2 (SARS-CoV-2). Five mutations (T27F, K31Y, L79W, R273Q, and N330Y) were introduced into the ACE2 domain to enhance its binding affinity and neutralizing activity against a broad range of SARS-CoV-2 variants, including the currently circulating JN.1 variant. The TNFR2 and GP130 domain confer strong binding to TNF and IL-6R-IL-6 complex, respectively, thereby effectively blocking pro-inflammatory signaling pathways. In a mouse model of acute lung inflammation induced by R848, treatment with the bifunctional and trifunctional fusion proteins markedly attenuated pulmonary pathology by dampening IL-6– and TNF–mediated inflammation. These findings demonstrate a promising therapeutic strategy for severe COVID-19 and offer a framework for designing multifunctional biologics against emerging viral infections.

**Supplementary Information:**

The online version contains supplementary material available at 10.1186/s43556-025-00320-4.

## Introduction

Despite a significant decrease in overall case numbers, severe COVID-19 continues to cause high morbidity and mortality, especially among the elderly and those with underlying health conditions such as diabetes, hypertension, obesity, or cardiovascular disease [1–3]. High viral loads and elevated levels of inflammatory cytokines are major contributors to severe disease in COVID-19. Excessive viral replication can trigger hyperinflammatory responses, resulting in tissue damage and systemic complications. The hyperinflammatory responses bears similarities to the cytokine release syndrome (CRS) frequently caused by CAR-T therapy. Both of these conditions are linked to the most severe and life-threatening toxicities [4]. Without timely and effective treatment, critical COVID-19 patients may progress to acute respiratory distress syndrome (ARDS), lung injury, multiple organ failure, or even death [5, 6]. Current therapeutic strategies primarily rely on monofunctional agents targeting either viral replication or inflammatory pathways. Therefore, therapeutic strategies that simultaneously reduce viral burden and dampen inflammatory cytokine levels would provide a highly effective approach for managing severe COVID-19.

SARS-CoV-2 enters host cells by binding the receptor-binding domain (RBD) of its spike (S) protein to angiotensin-converting enzyme 2 (ACE2) [7]. Current neutralizing antibodies and vaccines primarily target this interaction, but their efficacy is compromised by immune-evasive variants (e.g., Delta, Omicron) [8–10], and limited cross-serotype protection [11]. Soluble ACE2 has shown promise in clinical trials for circumventing viral escape mutations [12]. Previously studies have demonstrated that engineered ACE2-Fc fusion proteins exhibit therapeutic and protective effects in COVID-19 infection models, effectively neutralizing the virus in vivo with an acceptable safety profile [13–18].

Hyperinflammation in COVID-19 (termed cytokine storm or SIRS) involves excessive immune cell activation, leading to massive release of inflammatory factors including IL-6, TNF and IFN-γ [19–21]. Serum IL-6 and TNF levels independently predict disease severity and mortality, suggesting therapeutic potential in suppressing these cytokines [22, 23]. Current TNF inhibitors include neutralizing antibodies (e.g., adalimumab) and decoy receptors (e.g., etanercept); IL-6 inhibitors comprise ziltivekimab (IL-6-neutralizing antibody), tocilizumab (IL-6R-neutralizing antibody), and Olamkicept (soluble GP130-Fc fusion protein targeting the IL-6/IL-6R complex, under clinical evaluation). Antibodies targeting IL-6/IL-6R block both the pro-inflammatory and anti-inflammatory pathways associated with IL-6, whereas soluble GP130 (sGP130) only blocks its pro-inflammatory pathway [24–26]. This mechanistic insight underscores the superior therapeutic potential of sGP130-Fc.

In this study, we designed bifunctional and trifunctional fusion proteins utilizing decoy receptors capable of trapping both the virus and inflammatory cytokines. Specifically, the extracellular domain of ACE2 was linked to the N-terminus of human IgG1 Fc region, with GP130 and/or TNFR2 cytokine receptors fused to its C-terminus. These fusion proteins demonstrated potent neutralizing activity against multiple SARS-CoV-2 variants and effectively inhibited TNF and/or IL-6 signaling in vitro. Furthermore, they showed therapeutic efficacy in alleviating acute pneumonia injury in vivo. These findings establish a versatile platform for developing multifunctional biologics against severe COVID-19 and other inflammatory viral diseases.

## Results

### Design and characterization of bifunctional fusion proteins

**Fig. 1:**
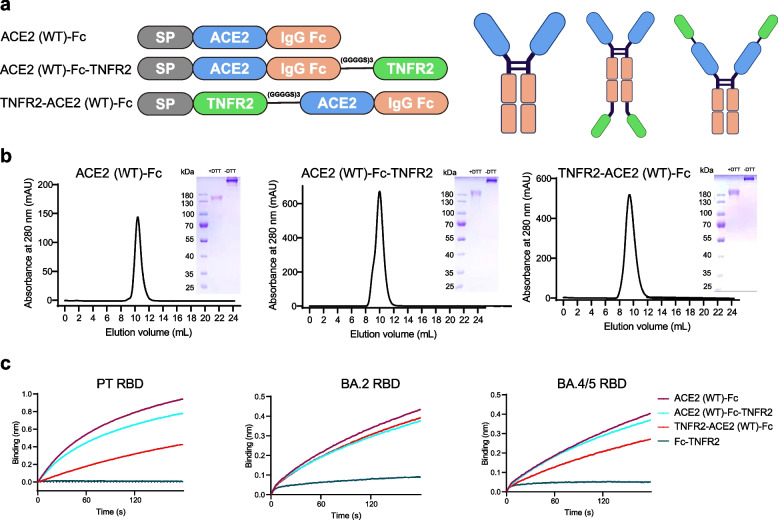
Design and characterization of bifunctional fusion proteins.** a** Schematic representation of the domain structure of fusion proteins ACE2 (WT)-Fc, ACE2 (WT)-Fc-TNFR2, and TNFR2-ACE2 (WT)-Fc. The domains are color-coded as follows: gray, mouse immunoglobulin κ light-chain signal peptide (SP); blue, extracellular domain of ACE2; orange, Fc domain of human IgG1; green, extracellular domain of TNFR2. This figure was created using BioRender with a valid license (https://www.biorender.com/, License ID: 665,857,182). **b** Fusion protein: ACE2 (WT)-Fc (left), ACE2 (WT)-Fc-TNFR2 (middle), and TNFR2-ACE2 (WT)-Fc (right), were purified using a Superdex™ 200 Increase 10/300 GL column. Protein purity was assessed by SDS-PAGE under reducing and non-reducing conditions. **c** Binding kinetics of fusion proteins to the RBDs from SARS-CoV-2 PT (left), BA.2 (middle), BA.4/5 (right) were measured using BLI

To develop bifunctional fusion proteins, two constructs were engineered by fusing wild-type (WT) ACE2 extracellular domain, human IgG1 Fc, and TNFR2 extracellular domain using a flexible linker (GGGGS)_3_: ACE2 (WT)-Fc-TNFR2 and TNFR2-ACE2 (WT)-Fc (Fig. 1a). These proteins were expressed in Expi293 cells and subsequently purified using Protein A affinity chromatography. Size exclusion chromatography was further performed to obtain fusion proteins with > 95% purity, as confirmed by SDS-PAGE under both reducing (+ DTT) and non-reducing (-DTT) conditions (Fig. 1b). The yields of both proteins were approximately 8 mg/L. Biolayer interferometry (BLI) was employed to determine the binding of the ACE2 domain in the fusion proteins to the SARS-CoV-2 RBDs. Compared to ACE2 (WT)-Fc, ACE2 (WT)-Fc-TNFR2 showed a slightly reduced binding affinity to the prototype (PT) RBD, while TNFR2-ACE2 (WT)-Fc exhibited a more significant decrease in affinity (Fig. 1c). A similar trend was detected for binding to BA.4/5 RBD, whereas no difference in binding was observed for BA.2 RBD between the two fusion proteins (Fig. 1c). These findings suggest that ACE2 (WT)-Fc-TNFR2 adopts a more favorable conformation and was thus selected for further investigations.

### Angiotensin-converting enzyme 2 mutant enhances binding affinity to SARS-CoV-2 variant receptor-binding domain

**Fig. 2:**
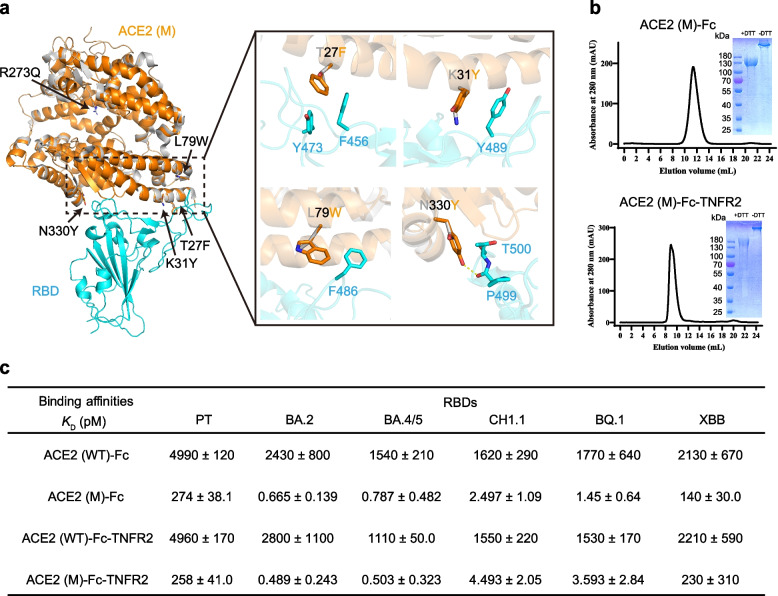
The ACE2 mutant exhibits increased binding affinity to SARS-CoV-2 RBDs.** a** Structural comparison of WT ACE2 (gray) and mutant ACE2 (orange) in complex with the PT RBD (cyan), based on PDB: 6lzg. Key interactions involving ACE2 mutations are highlighted. **b** Fusion proteins ACE2 (M)-Fc (top) and ACE2 (M)-Fc-TNFR2 (bottom) were purified using a Superdex™ 200 increase 10/300 GL column. Protein purity was assessed by SDS-PAGE under reducing and non-reducing conditions. **c** SPR analysis of the binding affinity between the fusion proteins and the RBDs from SARS-CoV-2 PT and its variants (BA.2, BA.4/5, CH1.1, BQ.1, XBB). The fusion proteins were immobilized on protein A chip and the *K*_D_ value for each paired interaction was calculated by single-cycle kinetics. The *K*_D_ values were shown as mean ± s.d. in triplicate

To enhance the neutralizing activity of the fusion protein against SARS-CoV-2, five specific amino acid substitutions, including T27F, K31Y, L79W, R273Q, and N330Y, were introduced into the ACE2 portion of the fusion protein ACE2 (WT)-Fc-TNFR2, based on insights from previous studies [27, 28]. These mutations were selected to simultaneously increase the binding affinity between ACE2 and the RBD and reduce the potential enzymatic side effects of ACE2 in future therapeutic applications. Among these mutations, the R273Q mutation aims to abolish the enzyme activity of ACE2. The remaining mutations T27F, K31Y, L79W, and N330Y were predicted to increase the hydrophobic interactions and hydrogen bonds between RBD and ACE2 (Fig. 2a). Specifically, ACE2 Y31 is predicted to form a π-π interaction with RBD Y489. ACE2 W79 may form stronger hydrophobic interactions with RBD F486 than L79. A hydrogen bond is predicted between ACE2 Y330 and RBD P499. This engineered ACE2 mutant was designated as ACE2 (M).

The ACE2 (M)-Fc-TNFR2 and ACE2 (M)-Fc proteins were successfully purified (Fig. 2b), and their binding affinities to RBDs were assessed using surface plasmon resonance (SPR). The results revealed that these mutations significantly enhanced the binding affinity of ACE2 to RBDs across multiple variants, including PT, BA.2, BA.4, CH1.1, BQ.1, and XBB (Fig. 2c and S1). Notably, ACE2 (WT)-Fc-TNFR2 exhibited a strong binding affinity to these RBDs at the nanomolar level, with dissociation constant (*K*_D_) values varying from 1.53 nM to 4.96 nM, which is consistent with previous reports [29]. While ACE2 (M)-Fc-TNFR2 achieved picomolar-range affinity, reaching the upper limit of detection (Fig. 2c and S1). Importantly, similar binding profiles were observed between ACE2 (WT)-Fc and ACE2 (WT)-Fc-TNFR2, as well as between ACE2 (M)-Fc and ACE2 (M)-Fc-TNFR2. Together, ACE2 (M)-Fc-TNFR2 was structurally optimized to increase RBD binding.

### ACE2 (M)-Fc-TNFR2 exhibits potent neutralizing activity against SARS-CoV-2 variants

**Fig. 3:**
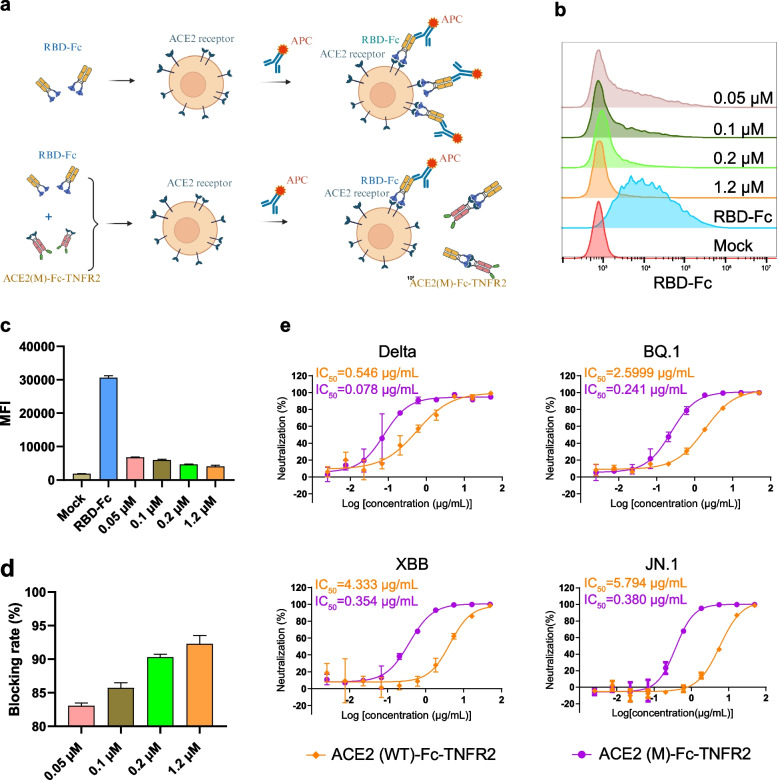
ACE2 (M)-Fc-TNFR2 exhibits potent neutralizing activity against SARS-CoV-2 variants.** a** Schematic diagram of ACE2 (M)-Fc-TNFR2 blocks the interaction between the RBD and the ACE2 receptor on Expi293F cells, as determined by flow cytometry. This figure was created using BioRender. **b-d** Binding of PT RBD-Fc to Expi293F-ACE2 cells was analyzed by flow cytometry. After incubation of the fusion protein with RBD-Fc at different concentrations (0.05 μM, 0.1 μM, 0.2 μM and 1.2 μM), the binding of RBD to Expi293F-ACE2 cells was detected using an anti-human IgG Fc-APC antibody (**b**). The binding amount was determined (**c**) and the blocking rate of RBD binding to ACE2 receptor by different concentrations of ACE2 (M)-Fc-TNFR2 was calculated by fluorescence intensity (**d**). **e** Neutralization of fusion proteins against SARS-CoV-2 Delta, BQ.1, XBB, and JN.1 pseudoviruses, and the IC_50_ values were determined by inhibition curves

Based on the enhanced affinity of ACE2 (M)-Fc-TNFR2, we subsequently evaluated its ability to inhibit the interaction between SARS-CoV-2 RBD and cell-surface ACE2 using a flow cytometry assay (Fig. 3a). Expi293F cells transiently expressing ACE2 were incubated with a series of pre-mixed solutions containing RBD-Fc and increasing concentrations of ACE2 (M)-Fc-TNFR2. RBD-Fc binding to ACE2 was detected using an APC-conjugated anti-human IgG antibody, and quantified by mean fluorescence intensity (MFI). A dose-dependent reduction in APC signal indicated competitive inhibition of RBD-Fc binding by ACE2 (M)-Fc-TNFR2 (Fig. 3b-3c). The blocking efficiency was calculated as [(MFI without inhibitor − MFI with inhibitor)/MFI without inhibitor] × 100%. These results confirm that ACE2 (M)-Fc-TNFR2 effectively blocks the interaction between RBD and membrane-bound ACE2 (Fig. 3d).

We further evaluated the neutralizing activity of the fusion protein using a vesicular stomatitis virus (VSV) pseudovirus-based neutralization assay (Fig. 3e). The results showed that the half-maximal inhibitory concentration (IC_50_) values of ACE2 (WT)-Fc-TNFR2 for neutralizing the Delta, BQ.1, XBB, and JN.1 pseudoviruses were 0.546, 2.599, 4.333, and 5.794 μg/mL, respectively. In contrast, the IC_50_ values of ACE2 (M)-Fc-TNFR2 were 0.078, 0.241, 0.354, and 0.380 μg/mL, respectively. Compared with ACE2 (WT)-Fc-TNFR2, ACE2 (M)-Fc-TNFR2 exhibited approximately 7.0-, 10.8-, 12.2-, and 15.2-fold higher neutralizing activity against the Delta, BQ.1, XBB, and JN.1 SARS-CoV-2 pseudoviruses, respectively. Collectively, these findings indicate that our strategy of modifying ACE2 with mutations was effective, endowing ACE2 (M)-Fc-TNFR2 with stronger neutralizing activity.

### ACE2 (M)-Fc-TNFR2 binds effectively to TNF

**Fig. 4:**
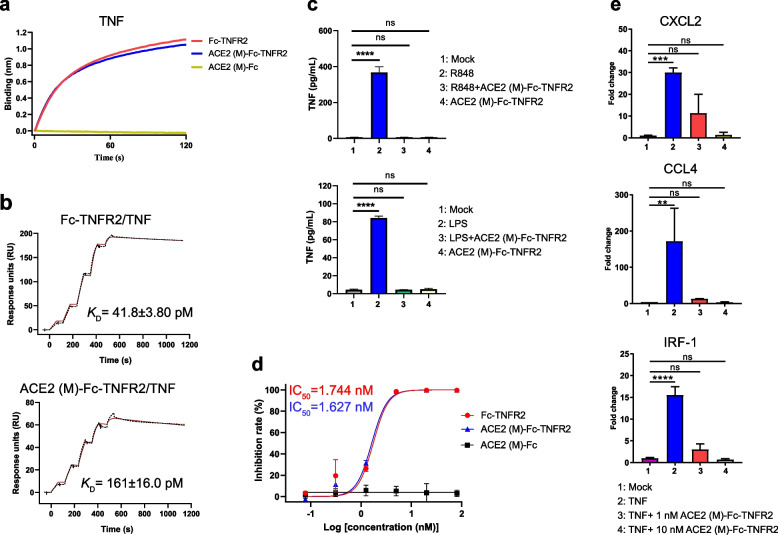
ACE2 (M)-Fc-TNFR2 effectively neutralizes TNF and inhibits downstream signaling.** a** The binding of fusion proteins to TNF was measured using BLI. **b** The binding affinity of fusion proteins to TNF were analyzed by SPR. **c** ACE2 (M)-Fc-TNFR2 was added to the supernatant of R848- (top) or LPS-(bottom) stimulated THP-1 cells, the TNF levels in the supernatant were measured using cytometric bead array (CBA) kit. **d** The 293 T NF-κb luciferase reporter cell line was stimulated after co-incubation of fusion proteins with TNF. The inhibition rate of the proteins was calculated based on the fluorescence intensity. **e** The THP-1 cells were stimulated after co-incubation of 1 nM and 10 nM ACE2 (M)-Fc-TNFR2 with TNF. Total RNA was extracted, and mRNA expression of *CXCL2* (top), *CCL4* (middle), and *IRF-1* (bottom) mRNA was quantified by qRT-PCR. Fold Change of genes was calculated as 2^−ΔΔCt^ and normalized to *GAPDH*. Statistical significance is denoted as follows: **P < 0.01, ***P < 0.001, ****P < 0.0001, and ns *P* ≥ *0.05*

After confirming the characterization of the ACE2 domain within the bifunctional fusion protein, we further validated the functionality of its TNFR2 region. The binding of ACE2 (M)-Fc-TNFR2 to TNF was evaluated using BLI. The fusion protein exhibited comparable TNF binding to TNFR2-Fc at 200 nM, whereas no interaction was detected for ACE2 (M)-Fc lacking the TNFR2 domain (Fig. 4a). An SPR assay was further performed to accurately determine the binding affinity between ACE2 (M)-Fc-TNFR2 and TNF. The results revealed that ACE2 (M)-Fc-TNFR2 strongly binds to TNF, with a *K*_D_ value of 161 pM, comparable to that reported in previous studies [30]. Although the affinity is slightly lower than that of Fc-TNFR2, it still exhibits high-affinity binding (Fig. 4b).

We next assessed the binding ability of ACE2 (M)-Fc-TNFR2 to bind TNF in the THP-1 cells. Stimulation of Tokyo human acute promyelocytic leukaemia-1 (THP-1) cells with lipopolysaccharide (LPS) or resiquimod (R848), which are agonists of Toll-like receptor 4 (TLR4) and Toll-like receptors 7/8 (TLR7/8), respectively, induces the production of pro-inflammatory cytokines, such as TNF and IL-6. Upon stimulation with LPS or R848 for 6 h, a significant increase in TNF levels was observed in the supernatant of THP-1 cells. However, when ACE2 (M)-Fc-TNFR2 was added to the cell supernatant, TNF levels were significantly decreased, reaching levels comparable to those in unstimulated control cells (Fig. 4c). These results demonstrate that ACE2 (M)-Fc-TNFR2 can effectively neutralize TNF secreted by cells.

### ACE2 (M)-Fc-TNFR2 effectively neutralizes TNF and inhibits downstream signaling

To assess the TNF neutralizing activity of the fusion proteins, quantitative experiments were conducted. The 293 T-NF-κB-Luc2 cell line enables monitoring of the NF-κB pathway through luciferase activity. Activation of the NF-κB pathway in this cell line by TNF can be quantified based on the levels of luciferase activity. Following a 10-min incubation with a gradient dilution of ACE2 (M)-Fc-TNFR2 and TNF, the mixture was added to the cells and further incubated for 6 h. The results demonstrated that ACE2 (M)-Fc-TNFR2 displayed the ability to neutralize TNF, with IC_50_ values of 1.627 nM, comparable to that of Fc-TNFR2 (Fig. 4d), indicating that the fusion with ACE2 does not impair the activity of TNFR2 domain. However, the *K*_D_ values for the two proteins measured by SPR showed a 3.85-fold difference, which is likely attributable to methodological variations in the detection assays.

Additionally, the potential impact of ACE2 (M)-Fc-TNFR2 on the downstream signaling pathway of TNF was assessed using quantitative reverse transcription polymerase chain reaction (qRT-PCR). THP-1 cells were incubated with mixtures containing TNF (10 ng/mL) and varying doses of ACE2 (M)-Fc-TNFR2 (1 nM and 10 nM) for 5 min, followed by a 16-h incubation period. The cells were then harvested and subjected to qRT-PCR analysis using specific primers to assess the expression levels of *C-X-C motif chemokine ligand 2 (CXCL2)*, *C–C motif chemokine ligand 4* (*CCL4)*, and *interferon regulatory factor 1* (*IRF-1)*. The results demonstrate a dose-dependent reductions in mRNA levels of *CXCL2*, *CCL4*, and *IRF-1*. Notably, TNF significantly increased the expression of *CXCL2* (30.0-fold), *CCL4* (172.1-fold), and *IRF-1* (15.6-fold) when compared to the *glyceraldehyde-3-phosphate dehydrogenase* (*GAPDH)* housekeeping gene as a reference control. However, the expression levels of *CXCL2*, *CCL4*, and *IRF-1* in either 1 nM or 10 nM ACE2 (M)-Fc-TNFR2 were gradually decreased and finally similar to that of mock cells (Fig. 4e). Taken together, these findings suggest that ACE2 (M)-Fc-TNFR2 can effectively neutralize TNF activity within the NF-κB pathway.

### ACE2 (M)-Fc-GP130 fusion proteins effectively bind Hyper-IL-6 

**Fig. 5:**
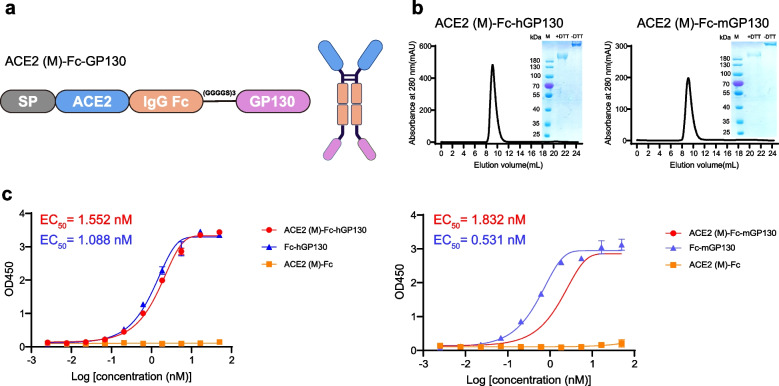
Design and characterization of bifunctional fusion proteins ACE2 (M)-Fc-GP130.** a** Schematic representation of the domain structure of fusion proteins ACE2 (M)-Fc-GP130 protein. This figure was created using BioRender. **b** Purification of ACE2 (M)-Fc-hGP130 (left) or ACE2 (M -Fc-mGP130 (right) protein. **c** The binding affinities of ACE2 (M)-Fc-hGP130 and ACE2 (M)-Fc-mGP130 to human (left) and mouse (right) Hyper-IL-6 were measured using ELISA, respectively

Given the role of IL-6 in the cytokine storm associated with COVID-19 and the successful validation of ACE2 (M)-Fc-TNFR2, we fused the GP130 to the C-terminus of ACE2 (M)-Fc, creating ACE2 (M)-Fc-GP130 (Fig. 5a). Since human IL-6 can activate cells from both human and murine sources, whereas murine IL-6 is species-specific [31, 32], we generated two constructs: ACE2 (M)-Fc-hGP130 and ACE2 (M)-Fc-mGP130, using human and mouse GP130, respectively, for functional evaluation in both species. In contrast, human TNFR2 can bind to murine TNF, making ACE2 (M)-Fc-TNFR2 suitable for mouse models. ACE2 (M)-Fc-hGP130 and ACE2 (M)-Fc-mGP130 proteins were purified (Fig. 5b), with yields of approximately 8 mg/L. Subsequently, their binding capacity to human (or murine) Hyper-IL-6 was determined through enzyme-linked immunosorbent assay (ELISA). The half-maximal effective concentration (EC_50_) values for ACE2 (M)-Fc-hGP130 and ACE2 (M)-Fc-mGP130 were 1.552 nM and 1.832 nM, respectively, comparable to those of Fc-hGP130 and Fc-mGP130. These findings indicate that ACE2 (M)-Fc-GP130 retained its original binding capability (Fig. 5c).

### Bifunctional fusion proteins can effectively ameliorate acute pneumonia in mice

**Fig. 6:**
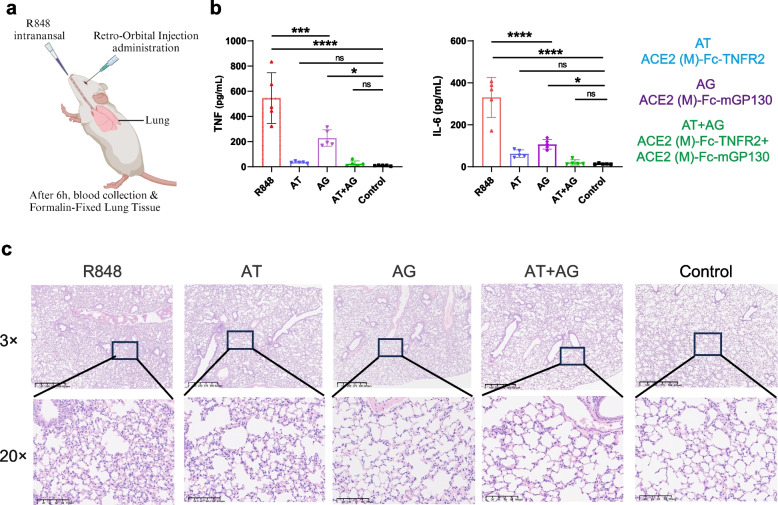
The bifunctional fusion proteins effectively ameliorate lung injury in mice.** a** Immunization strategy of the fusion proteins for treatment. BALB/c mice were administered R848 via intranasal administration. The fusion protein was injected via retro-orbital injection. This figure was created using BioRender. Mice were divided into three treatment groups: ACE2 (M)-Fc-TNFR2 (AT), ACE2 (M)-Fc-mGP130 (AG), and the combination group comprising ACE2 (M)-Fc-TNFR2 and ACE2 (M)-Fc-mGP130 (AT + AG). After 6 h, blood samples were collected, and the mice were euthanized for lung tissue fixation in 10% formalin. **b** Serum levels of TNF (left) and IL-6 (right) were measured with mouse CBA kit. **c** Lung tissues were stained with H&E staining to assess pathological changes. Statistical significance is denoted as follows: *P < 0.05, ***P < 0.001, ****P < 0.0001, and ns *P* ≥ *0.05*

To assess the protective efficacy of ACE2 (M)-Fc-TNFR2 and ACE2 (M)-Fc-mGP130, an acute inflammatory lung injury model was initially established in 6–8-week-old BALB/c mice via nasal administration of 2.5 mg/kg R848. Concurrently, the 300 μg of each fusion protein was administered through retro-orbital intravenous injection. After six hours, blood samples were collected and the mice were euthanized. The lung tissues were fixed using 10% formalin (Fig. 6a). Cytometric bead array (CBA) analysis revealed a significant elevation in serum TNF and IL-6 levels at 6 h post-R848 stimulation (Fig. 6b). In the ACE2 (M)-Fc-TNFR2 group and the combination treatment group, these cytokine levels returned to baseline, whereas the ACE2 (M)-Fc-mGP130 group showed a moderate increase relative to untreated controls (Fig. 6b). Histological examination demonstrated markedly inflammatory cell infiltration and alveolar wall thickening after R848 stimulation. Both single‑agent treatments exhibited a certain degree of lung protection. However, the combination of ACE2 (M)-Fc-TNFR2 and ACE2 (M)-Fc-mGP130 demonstrated superior efficacy in reducing lung inflammation compared with monotherapy with either fusion protein, resembling characteristics observed in healthy lungs (Fig. 6c).

### ACE2 (M)-Fc-hGP130-TNFR2 achieves efficacy equivalent to bispecific combination

**Fig. 7:**
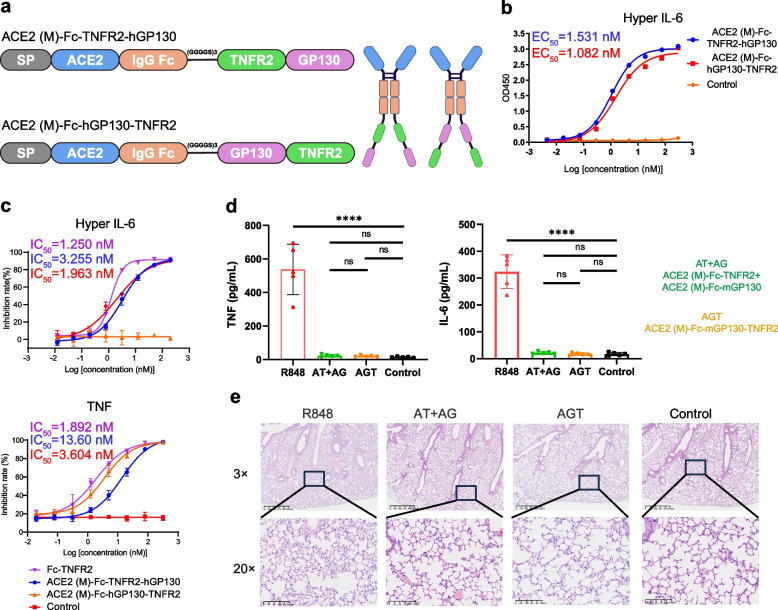
Design and characterization of trispecific fusion proteins.** a** Schematic representation of the domain structure of ACE2 (M)-Fc-TNFR2-hGP130 and ACE2 (M)-Fc-hGP130-TNFR2. This figure was created using BioRender. **b** The binding affinities of ACE2 (M)-Fc-TNFR2-hGP130 and ACE2 (M)-Fc-hGP130-TNFR2 to human Hyper-IL-6 was measured by ELISA. **c** The inhibitory effect of the fusion protein on human Hyper-IL-6 (left) and TNF (right) was determined using 293 T-STAT3-Luc2-Cell and 293 T-NF-κb-luciferase cell, respectively. **d** Similar to Fig. 5, serum levels of TNF (left) and IL-6 (right) were measured with mouse CBA kit. **e** Lung tissues were stained with H&E to assess pathological changes. Statistical significance is denoted as follows: ****P < 0.0001 and ns *P* ≥ *0.05*

In view of the efficacy of ACE2 (M)-Fc-TNFR2 and ACE2 (M)-Fc-mGP130 combination therapy and its promising clinical potential, we fused hGP130 and TNFR2 together with ACE2 (M)-Fc as a trifunctional fusion protein. Similar to the previous design, two fusion proteins — ACE2 (M)-Fc-hGP130-TNFR2 and ACE2 (M)-Fc-TNFR2-hGP130 — were engineered (Fig. 7a). The yields of both proteins were approximately 6 mg/L. The ELISA results showed no significant difference in the binding ability of the two fusion proteins towards human Hyper-IL-6 (Fig. 7b), which was consistently observed in their ability to neutralize and inhibit human Hyper-IL-6 activity as demonstrated by the 293 T-STAT3-Luc2 cell assay (Fig. 7c). Moreover, ACE2 (M)-Fc-hGP130-TNFR2 exhibited a slight reduction in TNF neutralization capacity, approximately twofold lower than that of TNFR2-Fc. However, ACE2 (M)-Fc-TNFR2-hGP130 demonstrated a significantly diminished TNF neutralization capacity, approximately 3.77-fold lower (Fig. 7c). This disparity may arise from the substantial difference in molecular weight between hGP130 and TNFR2, potentially leading to partial steric hindrance of TNFR2 by hGP130.

To evaluate in vivo efficacy, ACE2 (M)-Fc-mGP130-TNFR2 was assessed in an R848-induced acute lung injury model, with the combination therapy of ACE2 (M)-Fc-TNFR2 (AT) and ACE2 (M)-Fc-mGP130 (AG) serving as a comparator. Serum levels of TNF and IL-6 did not differ significantly among the trispecific fusion group, the combination group, and untreated controls (Fig. 7d). Histological analysis revealed that both treatment groups effectively mitigated pulmonary inflammation, with lung tissue architecture closely resembling that of healthy mice (Fig. 7e). The serum biochemistry tests showed that despite an elevated alanine aminotransferase (ALT) levels in both R848-stimulated and AGT-treated groups, all values remained within the normal range (Fig. S2a). No significant differences in creatinine (CREA) level were observed among all groups (Fig. S2b). These results indicate preserved normal hepatic and renal function in the subjects. In addition, hematoxylin and eosin (H&E) staining of the heart, liver, spleen, and kidney showed no significant pathological changes across all groups (Fig. S2c). These findings demonstrate that the trispecific fusion protein ACE2 (M)-Fc-mGP130-TNFR2 achieves therapeutic efficacy comparable to that of the bispecific combination therapy with a favorable safety profile, thereby validating its potential as a therapeutic alternative.

## Discussion

Despite the development of vaccines, monoclonal antibodies, and small molecule drugs, COVID-19 continues to pose a significant public health challenge, particularly to individuals with underlying health conditions who are more susceptible to severe disease and mortality. The persistent emergence of SARS-CoV-2 variants that show significant immune escape and induce cytokine storms further complicates the situation. In terms of antivirals, although antibody-based therapeutics, including monoclonal antibodies and cocktail therapies, have demonstrated effectiveness, antibody-resistant viral variants limit their long-term efficacy [33–35]. Small-molecule drugs targeting viral RNA-dependent RNA polymerase, such as remdesivir and molnupiravir, have shown some therapeutic efficacy. However, they are mainly administered to patients with early-stage or moderate infections, and widespread use can easily lead to the emergence of drug resistance [36, 37]. In contrast, as the cellular receptor mediating SARS-CoV-2 entry, utilizing ACE2 as a decoy receptor can effectively circumvent immune evasion of neutralizing antibodies by viral variants. In this study, we introduced three additional mutations (K31Y, L79W, and N330Y) to the previously designed double mutant (T27F, R273Q) [27]. The results of this study demonstrate that these modifications further enhanced the affinity of ACE2 for the viral spike protein and its neutralizing potency against the virus.

In parallel, the current inhibition of cytokines primarily involves biological agents such as soluble receptors and neutralizing antibodies. It is worth noting that such neutralizing antibodies may elicit immunogenic responses in diverse individuals [38]. Therefore, we explored the use of soluble cytokine decoy receptors as a potentially safer and more broadly applicable alternative. Of the two TNF receptors, TNFR2 was selected for its anti-inflammatory properties and its established therapeutic value in autoimmune conditions such as rheumatoid arthritis [39]. Consistent with these properties, our results confirmed a favorable efficacy in neutralizing TNF. Among the IL-6 receptors, soluble GP130 selectively inhibits IL-6 trans-signaling — which is implicated in driving inflammation — while sparing classical signaling pathways that mediate anti-inflammatory and regenerative responses, and has shown potential therapeutic efficacy in various inflammatory diseases [40–42].

Bifunctional fusion proteins have achieved significant success as targeted therapeutic agents in the fields of oncology and autoimmune diseases, where they enhance efficacy through synergistic action on multiple targets. However, no bifunctional fusion protein drugs have yet been approved for viral infectious diseases. In this study, we employed a bifunctional fusion protein composed of soluble decoy receptors as a therapeutic platform, aiming to provide an alternative with enhanced safety and broad-spectrum applicability compared with neutralizing antibodies. Specifically, we developed a bifunctional fusion protein strategy targeting critically ill COVID-19 patients — integrating a viral entry receptor decoy (ACE2) with immune-modulatory domains (e.g., TNFR2 and GP130) — designed to simultaneously neutralize the virus and modulate cytokines. This platform can be easily applied to other existing or emerging coronaviruses with zoonotic potential that rely on ACE2 as the receptor to infect the host, including SARS-CoV, HCoV-NL63, and various bat coronaviruses [43–45]. Moreover, the modular nature of the platform allows the ACE2 domain to be replaced with other viral receptor domains or pathogen-specific neutralizing antibody fragments. It also allows the TNFR2 and GP130 domains to be substituted for other proteins with the potential to regulate inflammatory storms (such as CD24 or TLR1) to address different immunopathology. This opens avenues for the development of similar bifunctional or trispecific fusion proteins against other respiratory pathogens, such as influenza virus or respiratory syncytial virus (RSV), which also induce cytokine-driven immunopathology.

In this study, the anti-inflammatory activities of the GP130 and TNFR2 domains were preliminarily validated in an acute inflammatory murine model. However, the functional contribution and immunogenic profile of the ACE2 (M) moiety require further evaluation. Subsequent investigations need to be conducted in more animal models — e.g., hACE2 transgenic mice or non-human primates — to perform a systematic evaluation of this bifunctional fusion protein. Specifically, the following objectives should be addressed: (i) confirmation of the ACE2 (M)’s capacity to competitively inhibit viral entry, and (ii) evaluating the efficacy of the GP130 and TNFR2 domains in neutralizing IL-6 and TNF by competing with endogenous receptors. Meanwhile, a comprehensive functional evaluation should be conducted to assess therapeutic efficacy against infections caused by SARS-CoV-2.

In conclusion, the multifunctional fusion proteins developed in this study demonstrate dual therapeutic efficacy by concurrently neutralizing multiple SARS-CoV-2 variants and mitigating cytokine storms primarily mediated by TNF/IL-6 proinflammatory cytokines. These findings establish a promising therapeutic approach for critically ill COVID-19 patients. The dual-mechanism cooperative treatment paradigm presents substantial clinical potential for viral diseases associated with severe inflammatory responses, warranting further investigation into its application for diverse viral infections.

## Materials and methods

### Construction, expression and purification of bifunctional fusion proteins

The cDNA sequences encoding the extracellular domains (ECDs) of human ACE2 (residues 18–740, GenBank: BAB40370.1), TNFR2 (residues 23–257, GenBank: NP_001057.1), human GP130 (residues 23–619, GenBank: AAA59155.1), mouse GP130 (residues 23–617, GenBank: AAH58679.1), human IL-6Rα (residues 20–355, GenBank: KAI4082890.1), human IL-6 (residues 29–211, GenBank: AFF18412.1), mouse IL-6Rα (residues 20–354, GenBank: CAA37810.1) and mouse IL-6 (residues 25–211, GenBank: ABG81953.1) were codon-optimized and chemically synthesized by Logenbio. For expression constructs, the ACE2 ECD was subcloned into the pCAGGS vector containing an N-terminal Igκ signal peptide and a C-terminal human IgG1 Fc tag using the restriction enzymes *BsiwI* and *BstbI*, generating pCAGGS-ACE2(WT)-Fc. To generate bifunctional fusion proteins, the TNFR2 ECD was connected to either the N- or C-terminus of ACE2 (WT)-Fc through a flexible (GGGGS)_3_ linker, resulting in pCAGGS-TNFR2-ACE2 (WT)-Fc and pCAGGS-ACE2 (WT)-Fc-TNFR2 constructs, respectively. We subsequently introduced five key mutations (T27F, K31Y, R273Q, L79W, and N330Y) into the ACE2 domain using site-directed mutagenesis. Following the same cloning strategy, we generated the corresponding mutant constructs: pCAGGS-ACE2 (M)-Fc, pCAGGS-TNFR2-ACE2 (M)-Fc, and pCAGGS-ACE2 (M)-Fc-TNFR2. The TNFR2 segment in pCAGGS-ACE2 (M)-Fc-TNFR2 was replaced with human or mouse GP130, generating pCAGGS-ACE2 (M)-Fc-hGP130 and pCAGGS-ACE2 (M)-Fc-mGP130, respectively. Additionally, TNFR2 was inserted into pCAGGS-ACE2 (M)-Fc-hGP130 to produce pCAGGS-ACE2 (M)-Fc-TNFR2-hGP130, and human GP130 was introduced into pCAGGS-ACE2 (M)-Fc-TNFR2 to create pCAGGS-ACE2 (M)-Fc-GP130-TNFR2. Plasmids encoding human or murine Hyper-IL-6 were constructed by connecting the respective soluble IL-6 receptor (sIL-6R) to IL-6 via a GGGGSGGGGS linker peptide. The fusion genes were then cloned into the pCAGGS vector and a His-tag was fused at the C-terminus [46]. For cell surface expression studies, full-length ACE2 was cloned into the pCDH-EF1-T2A-copGFP lentiviral vector, transfected into target Expi293F cells and was identified by GFP fluorescence. All cell lines in this study were authenticated by short tandem repeat (STR) profiling and tested negative for mycoplasma.

The plasmids encoding the fusion proteins were transiently transfected into Expi293F cells for protein expression. Transfection was performed using polyethyleneimine (PEI) at a plasmid DNA-to-PEI ratio of 1:3 (w/w). Culture supernatants were harvested 5–7 days post-transfection, filtered through a 0.22 μm membrane, and subjected to affinity purification on a Protein A column. Further purification was carried out by size-exclusion chromatography (SEC) on a Superdex™ 200 Increase 10/300 GL column (GE Healthcare) equilibrated with PBS buffer (pH 7.4).

### Structural visualization in PyMOL

The structural visualization of ACE2-RBD complexes was performed by modifying the existing crystal structure (PDB: 6LZG) followed by molecular modeling and analysis using PyMOL.

### Octet binding assay

For binding affinity measurements, SARS-CoV-2 RBD with a C-terminal His tag (10 μg/mL) was immobilized on NTA biosensors. The loaded sensors were then incubated with 200 nM solutions of ACE2 (WT)-Fc, ACE2 (WT)-Fc-TNFR2, or TNFR2-ACE2 (WT)-Fc, respectively. Additionally, biotinylated TNF (10 μg/mL) was captured on streptavidin (SA) biosensors and subsequently combined with 200 nM solutions of TNFR2-Fc or ACE2 (WT)-Fc-TNFR2. For all experiments, the protein buffer consisted of PBS containing 0.05% (v/v) Tween 20. The binding interactions were assessed using Octet BLI assays on the Octet R2 system (ForteBio). The resulting data were analyzed using Octet BLI Analysis 12.2.1.3 software.

### SPR assay

The binding affinities between ACE2 fusion proteins and SARS-CoV-2 PT or variant RBDs were determined by SPR using a Biacore 8 K system (GE Healthcare). In single-cycle kinetics mode, ACE2 fusion proteins were captured on Protein A sensor chips. Serial dilutions of SARS-CoV-2 PT or variant RBDs were injected over the chip surface to assess their binding to each ACE2 fusion protein. All protein samples were prepared in PBS containing 0.05% (v/v) Tween 20 as running buffer. Binding kinetics were analyzed by globally fitting the sensor gram data to a 1:1 Langmuir binding model with Biacore Insight Evaluation Software (v3.0.12.15655) to determine the equilibrium dissociation constants (*K*_D_). The same analytical approach was applied to quantify TNF binding affinities for all fusion protein constructs.

### Flow cytometry

For receptor blocking assays, we first generated full-length ACE2-expressing Expi293F cells by transfecting with pCDH-EF1-ACE2 (WT)-T2A-copGFP plasmid construct. Successful transfection was confirmed by GFP fluorescence on the cell membrane surface. At 48 h post-transfection, pre-formed complexes of serially diluted ACE2 (M)-Fc-TNFR2 with 0.6 nM SARS-CoV-2 RBD (PT)-Fc were prepared by 10-min incubation on ice. These complexes were then added to 6 × 10^5^ cells per condition and allowed to bind for 20 min at 4 °C. After washing with ice-cold FACS buffer (PBS containing 2% (v/v) FBS), cells were stained with APC-conjugated goat anti-human IgG antibody (BioLegend, Cat# 410,711) for 40 min at 4 °C in the dark. Following two additional washes, fluorescence signals were acquired using a Beckman flow cytometer and analyzed with CytExpert software (v2.4).

### Cytokine assay

The TNF neutralization assay was performed using THP-1 cells plated at a density of 2 × 10^5^ cells per well in 24-well plates. In this experiment, three control groups were established: an untreated control group, an LPS/R848 stimulation group, and a control group with only fusion protein added. Cells in the experimental group were stimulated with 50 ng LPS/200 ng R848 combined with 5 μg ACE2 (M)-Fc-TNFR2. Following 5 h of incubation at 37 °C, the culture supernatants of each group were collected and incubated with Protein A magnetic beads for 1 h at 4 °C with gentle rotation. After centrifugation, demagnetized beads were discarded remove the captured TNF and fusion protein complexes. The concentration of TNF in the supernatants of each group was quantified using the BD CBA Human Inflammatory Cytokines Kit (BD Biosciences) according to the manufacturer's protocol, with fluorescence measurements acquired on a CytoFLEX flow cytometer (Beckman Coulter).

### Pseudovirus neutralization assay

We generated pseudotyped viruses presenting SARS-CoV-2 prototype or variant spike proteins using the replication-deficient vesicular stomatitis virus vector backbone (rVSV-∆G-GFP), as previously described [27]. In the neutralization assay, 50 μL of solution containing 50 μg of the fusion protein was pre-incubated with 1,000 transducing units (TU) of pseudovirus at 37 °C for 1 h. These mixtures were then added to confluent monolayers of Vero E6 cells (ATCC CCL-81) in 96-well plates. Following 15 h of incubation at 37 °C with 5% CO₂, viral transduction efficiency was quantified by enumerating GFP-positive cells using a CQ1 confocal quantitative image cytometer (Yokogawa Electric Corporation).

### Quantitative real-time polymerase chain reaction (qRT-PCR)

To evaluate the inhibitory effect of the fusion protein on TNF activity, THP-1 cells were seeded in 6-well plates at a density of 2 × 10^6^ cells per well. A mixture of 20 ng TNF and either 50 μL of 1 nM or 10 nM ACE2 (M)-Fc-TNFR2 was incubated for 5 min at room temperature prior to addition to the cells. Following treatment, plates were incubated for 16 h at 37 °C under 5% CO₂. Total RNA was isolated using TRIzol™ reagent (Invitrogen), and 1 µg of RNA was reverse-transcribed into cDNA using a High-Capacity cDNA Reverse Transcription Kit (TransGen Biotech, AT311-03). qRT-PCR was performed with SYBR Green qPCR Master Mix (Vazyme Biotech) according to the manufacturer’s protocol. Gene-specific primers (sequences provided in Table S1) were used to amplify target genes. Relative gene expression was calculated using the 2^−△△Ct^ method and was expressed as fold-change of gene expression normalized to *GAPDH*.

### Luciferase reporter assay

The NF-κB signaling activity was assessed using 293 T-NF-κB-Luc2 cell line (Kyinno) plated at a density of 2 × 10^4^ cells/well in 96-well culture plates. The fusion proteins (TNFR2-Fc, ACE2 (M)-Fc, and ACE2 (M)-Fc-TNFR2) were serially diluted four-fold from an 80 nM stock solution. Each diluted protein (50 μL) was pre-incubated with 50 μL of recombinant human TNF (final concentration: 10 ng/mL) for 5 min at 37 °C before adding to cells. Following 6 h of stimulation, luciferase activity was quantified using the SpectraMax i3x multimode microplate reader (Molecular Devices) with SoftMax Pro software (v7.0.3). Relative NF-κB activity was calculated by normalizing luminescence values (relative luminescence units, RLU) to untreated controls. The inhibition rate of the fusion protein was determined based on relative NF-κB activity.

The 293 T-STAT3-Luc2 cells were plated at a density of 2 × 10^4^ cells/well in 96-well plates. The fusion proteins (hGP130-Fc, ACE2 (M)-Fc, and ACE2 (M)-Fc-hGP130) were serially diluted four-fold from an 80 nM stock solution. Each diluted protein (50 μL) was pre-incubated with 50 μL of recombinant human Hyper-IL-6 (hIL-6Rα-G4S-hIL-6; final concentration 0.5 ng/mL) for 5 min at 37 °C to form inhibitor-cytokine complexes before addition to cells. Following 6 h of stimulation, luciferase activity was quantified using a SpectraMax i3x multimode microplate reader (Molecular Devices) with SoftMax Pro software (v7.0.3). Data were analyzed using the same normalization method as described for the TNF experiments.

### Enzyme-Linked Immunosorbent Assay (ELISA)

The binding affinity of fusion proteins to Hyper-IL-6 was evaluated by ELISA. Briefly, 96-well EIA/RIA plates (Corning®) were coated with 2 μg/mL of recombinant Hyper-IL-6-His protein in carbonate-bicarbonate buffer (pH 9.6) and incubated overnight at 4 °C. After three washes with PBST, plates were blocked with 5% non-fat dry milk in PBST at 37 ℃ for 1 h. Serial dilutions of fusion proteins (starting concentration: 50 nM) were prepared in blocking buffer and added to duplicate wells. Following 1 h incubation at 37 °C, plates were washed with PBST. Horseradish peroxidase (HRP)-conjugated goat anti-human IgG antibody (Boaosen Biotechnology, cat. no. bs-0297G; 1:5,000 dilution in PBS) was added and incubated for 1 h at 37 °C. After thorough washing, the wells were incubated with TMB substrate for 15 min, followed by the addition of 2 M H₂SO₄ to terminate the reaction. Absorbance was immediately measured at 450 nm using a microplate reader.

### Mouse experiments

Specific pathogen-free (SPF) female BALB/c mice (18–22 g), aged 6–8 weeks, were purchased from Beijing Vital River Laboratory Animal Technology Co., Ltd. (licensed by Charles River). The mice were housed in the laboratory animal facilities at the Institute of Microbiology, Chinese Academy of Sciences (IMCAS) under SPF conditions. Each cage contained five mice, which were provided with unrestricted access to water and a standard chow diet. The housing environment was maintained on a 12-h light/dark cycle, with a temperature range of 20–25 °C and Humidity of 40–70%. All animal experiments were approved by the Committee on the Ethics of Animal Experiments of IMCAS and conducted in accordance with the recommendations outlined in the Guide for the Care and Use of Laboratory Animals of the IMCAS Ethics Committee.

To induce acute lung inflammation, mice (n = 5 per group) received an intranasal administration of 50 µg R848, simultaneously with a retro-orbital injection of 300 µg (in 200 µL) of fusion protein. Control groups included an R848-only group and a PBS group. Treatment groups included: ACE2 (M)-Fc-TNFR2 (AT) group, ACE2 (M)-Fc-mGP130 (AG) group, combination (AT + AG) group (150 μg ACE2 (M)-Fc-TNFR2 and 150 μg ACE2 (M)-Fc-mGP130/200 μL), or ACE2 (M)-Fc-mGP130-TNFR2 (AGT) group. After six hours, blood samples were collected, and the mice were euthanized. Major organs (lung, heart, liver, spleen and kidney) were fixed with 4% paraformaldehyde and stained with H&E. The serum concentrations of TNF and IL-6 were measured using the BD CBA Mouse Inflammatory Cytokine Kit, following the manufacturer's instructions. Serum samples were sent to Servicebio for biochemical analysis, including the liver-function marker ALT and the kidney-function marker CREA.

### Statistical analysis

Data are presented as means ± standard deviations (SD). Statistical significance was determined by two-tailed unpaired Student's t-test for comparisons between two groups or one-way ANOVA for multiple group comparisons. Dose–response curves were fitted using nonlinear regression analysis (log[inhibitor] vs. response with variable slope) to calculate IC_50_ values. Statistical significance is denoted as follows: *P < 0.05, **P < 0.01, ***P < 0.001, ****P < 0.0001 and not significant (ns) *P* ≥ *0.05*.

## Supplementary Information

Supplementary Material 1.

## Data Availability

Data will be made available on request.
