# Supplementary material for: Development of a trispecific fusion protein based on angiotensin-converting enzyme 2, glycoprotein 130, and tumor necrosis factor receptor 2 as a promising therapeutic for COVID-19
Source: Mol Biomed. 2025 Oct 15;6:74. doi: 10.1186/s43556-025-00320-4 (PMC12521702; doi:10.1186/s43556-025-00320-4)
Supplement: Supplementary file 1 — Supplementary Material 1. [file 43556_2025_320_MOESM1_ESM.docx]

**Supplemen****tary material**

**Development of a trispecific fusion protein based on angiotensin-converting enzyme 2, glycoprotein 130, and tumor necrosis factor receptor 2 as a promising therapeutic for COVID-19**

Yongfeng Qiao^a,b^^,#^, Yanjun Han^a,b,#^, Lu Zhao^a,b,#^, Wenjing Gao^a,b^, Hong Hu^b,e^, Chao Su^c^, Anqi Zheng^d^, Junqing Sun^a,d^, Mingxiong Tian^c^, Yarong Wu^b^, Lianmei Bai^b^, Yuping Lei^f^, Jiahao Wu^a,b^, Weibing Zhang^a,b^, Pu Han^d^, Xiaoyu Li^b^, Chunbo Dong^a,b,*^, Haidong Wang^a,*^, Zhida Liu^a,b,g,*^, Pengcheng Han^c,*^

^a^College of Veterinary Medicine, Shanxi Agricultural University, Jinzhong, China

^b^Shanxi Academy of Advanced Research and Innovation, Taiyuan, China

^c^Jiangsu Provincial Key Laboratory of Critical Care Medicine, School of Medicine, Zhongda Hospital, Advanced Institute for Life and Health, Southeast University, Nanjing, 210009 China

^d^CAS Key Laboratory of Pathogen Microbiology and Immunology, Institute of Microbiology, Chinese Academy of Sciences, Beijing, China

^e^Ankerui (Shanxi) Biological Cell Co., Ltd., Taiyuan, China

^f^Shanxi Animal Disease Prevention and Control Center, Taiyuan, China

^g^MOE Key Laboratory of Coal Environmental Pathogenicity and Prevention, Shanxi Medical University, Taiyuan, China

^*^Correspondence:

Pengcheng Han ([101013216@seu.edu.cn](mailto:101013216@seu.edu.cn))

Zhida Liu ([zhida_liu@saari.org.cn](mailto:zhida_liu@saari.org.cn))

Haidong Wang ([wanghaidong@sxau.edu.cn](mailto:wanghaidong@sxau.edu.cn))

Chunbo Dong ([Chunbo_dong@saari.org.cn](mailto:Chunbo_dong@saari.org.cn))

^#^These authors contributed equally to this work

Table S1 qRT-PCR primers list

| Primer name | Sequence (5’-3’) |
| --- | --- |
| GAPDH-F | CCACCCAGAAGACTGTGGAT |
| GAPDH-R | GTTGAAGTCAGAGGAGACCACC |
| CCL4-F | AGGAAGCTTCCTCGCAACTT |
| CCL4-R | TCAGTTCAGTTCCAGGTCATACA |
| CXCL2-F | CTCAAGAATGGGCAGAAAGC |
| CXCL2-R | AAACACATTAGGCGCAATCC |
| IRF-1-F | CCACCCAGAAGACTGTGGAT |
| IRF-1-R | GTTGAAGTCAGAGGAGACCACC |

Table S2 List of Abbreviation

| Abbreviation | Definition |
| --- | --- |
| SARS-CoV-2 | Severe Acute Respiratory Syndrome Coronavirus 2 |
| COVID-19 | Coronavirus Disease 2019 |
| RSV | Respiratory Syncytial Virus |
| SIRS | Systemic Inflammatory Response Syndrome |
| ARDS | Acute Respiratory Distress Syndrome |
| GP130 | Glycoprotein 130 |
| TNFR2 | Tumor Necrosis Factor Receptor 2 |
| TNF | Tumor Necrosis Factor |
| IL-6 | Interleukin-6 |
| IL-6R | Interleukin-6 Recptor |
| hyper-IL-6 | IL-6R-GGGGSGGGGS-IL-6 |
| RBD | Receptor binding domain |
| BA.2 | SARS-CoV-2 Omicron sub-lineage BA.2 |
| BA.4/5 | SARS-CoV-2 Omicron sub-lineages BA.4 and BA.5 |
| CH1.1 | SARS-CoV-2 Omicron sub-variant CH.1.1 |
| BQ.1 | SARS-CoV-2 Omicron sub-variant BQ.1 |
| XBB | SARS-CoV-2 Omicron sub-variant XBB |
| ACE2 | Angiotensin-converting enzyme 2 |
| WT | Wild-type |
| PT | Prototype |
| MT | Monotherapy Group |
| CT | Combination Therapy Group |
| BLI | Biolayer interferometry |
| SPR | Surface Plasmon Resonance |
| VSV | Vesicular Stomatitis Virus |
| ELISA | Enzyme-Linked Immunosorbent Assay |
| GFP | Green Fluorescent Protein |
| LPS | Lipopolysaccharide |
| R848 | Resiquimod |
| EC_50_ | Half-maximal Effective Concentration |
| IC_50_ | Half-maximal Inhibitory Concentration |
| ALT | alanine aminotransferase |
| CREA | creatinine |


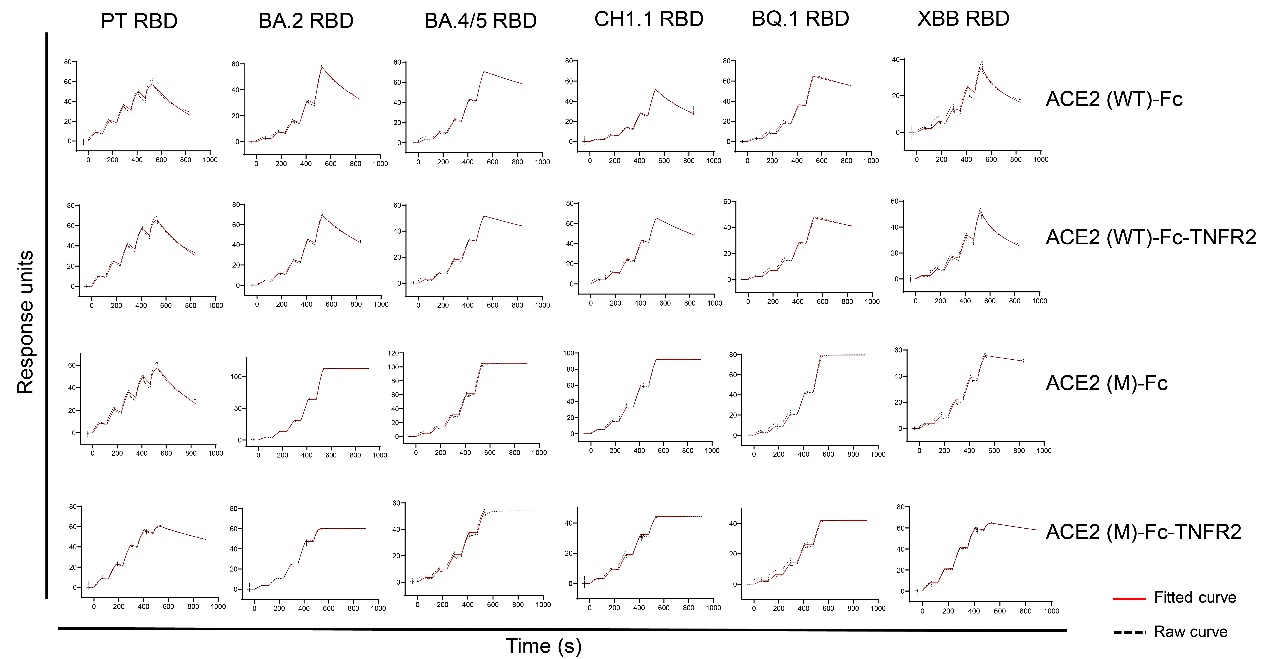
 **Fig. S1 SPR analysis of the binding affinity between the fusion proteins and SARS-CoV-2 RBDs.** The fusion proteins were immobilized on protein A chip and the *K*_D_ values with different concentrations of RBDs from SARS-CoV-2 PT or variants (BA.2, BA.4/5, CH1.1, BQ.1, XBB) were calculated by single-cycle kinetics.


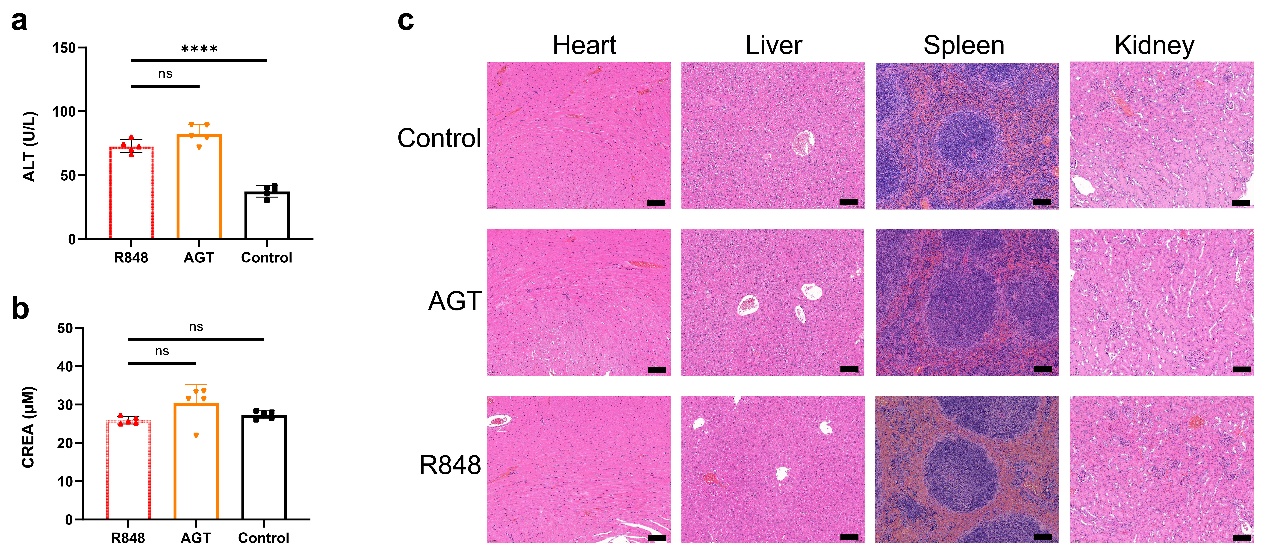
**Fig. S2 Safety assessment of the trispecific fusion protein ACE2(M)-Fc-mGP130-TNFR2 (AGT) *in vivo*.** The serum samples from control, R848 and AGT groups were analyzed for hepatic function biomarkers (ALT(**a**)) and renal function biomarker (CREA(**b**)). **c** H&E staining for the main organs, including heart, liver, spleen and kidney. Statistical significance is denoted as follows: *****P* < 0.0001 and ns *P* ≥ *0.05*. Scale bars, 100 μm
